# Supplementary material for: Disruption of normal saline supply chain due to a disaster: an analysis of the impact of normal saline shortage on anesthesia practice in a large hospital system and models toward resiliency
Source: Front Med (Lausanne). 2026 Jan 16;12:1671978. doi: 10.3389/fmed.2025.1671978 (PMC12855094; doi:10.3389/fmed.2025.1671978)
Supplement: Supplementary file 1 [file Table_1.DOCX]

Qualitative Survey Questions:

Survey only for those who were present during Hurricane Maria (2017-2018)

1. Has your care of patients in the OR been adversely affected by medication shortages in the past two years?
   1. Yes
   2. No
   3. How?
2. Which medications were on shortage that you felt most adversely affected you?
   1. Narcotic
   2. Bicarbonate
   3. NS
   4. Lidocaine
   5. Other
3. How often to you use the small saline bags (<500cc)?
   1. Daily
   2. Weekly
   3. Monthly
   4. Almost Never
4. What do you use the saline bags for?
   1. Insulin
   2. Ketamine
   3. Antibioitcs
   4. Other
5. What are your alternatives for using the small saline bags?
   1. D10W
   2. D5W
   3. D51/2NS
   4. D5LR
   5. Other
6. What is your alternative for compounding shortage medication?
   1. Syringe pumps
   2. Push-dose
   3. Syringe/aliquot dilution
   4. Other
7. Where do you obtain the small saline bags?
   1. Pharmacy
   2. OR
   3. Supply Room
   4. Called for by Tech
   5. Other
8. Where do you look for them if they are not in your default location?
   1. (Open question)
9. In what context do you use 0.9% Sodium Chloride (1 L bags)?
   1. Routine Crystalloid
   2. Carrier with Transfusion (Blood)
   3. Hyperkalemic Patients
   4. IV start
   5. Continued from pre-op
   6. Other
10. How often to you use 0.9% Sodium Chloride (1 L bags)?
    1. Daily
    2. Weekly
    3. Monthly
    4. Almost Never
11. Where do you obtain your 0.9% Sodium Chloride (1 L bags)?
    1. Pharmacy
    2. OR
    3. Supply Room
    4. Called for by Tech
    5. Other
12. What are your alternatives for using 0.9% Sodium Chloride (1 L bags)?
    1. 1L LR
    2. 1L Plasmalyte
    3. 1L D5 1/2NS
    4. Other
